# Pain management in hospitalized children: A cross-sectional study^*^

**DOI:** 10.1590/1980-220X-REEUSP-2022-0008en

**Published:** 2022-05-30

**Authors:** Joese Aparecida Carvalho, Danton Matheus de Souza, Flávia Domingues, Edgar Amatuzzi, Márcia Carla Morete Pinto, Lisabelle Mariano Rossato

**Affiliations:** 1 Universidade de São Paulo, Escola de Enfermagem, Departamento de Enfermagem Materno-Infantil e Psiquiátrica, São Paulo, SP, Brazil.; 2 Instituto Israelita de Ensino e Pesquisa Albert Einstein, São Paulo, SP, Brazil.

**Keywords:** Pain, Pain Management, Child Health, Pediatric Nursing, Dolor, Manejo del Dolor, Salud del Niño, Enfermería Pediátrica, Dor, Manejo da Dor, Saúde da Criança, Enfermagem Pediátrica

## Abstract

**Objective:**

To characterize pain management in hospitalized children.

**Method:**

This is an observational, cross-sectional, retrospective and descriptive study of quantitative approach, carried out in a secondary hospital in the city of São Paulo, through analysis of 1,251 medical records of children admitted to the pediatric department. Data were tabulated and analyzed through descriptive statistics.

**Results:**

A total of 88.8% of children were assessed for pain with standardized instruments and 86% had analgesia prescribed. Among the assessments, 37.8% of the children had pain; of these, 26% had severe pain, greater in orthopedic conditions; 18.3% were not medicated, even with the presence of pain and prescribed analgesia; 4.3% had no analgesics prescribed; only 0.4% received non-pharmacological measures, and 40.3% had a report of reassessment. Professionals provided greater analgesia to children with surgical and orthopedic conditions compared to clinical conditions (p < 0.05).

**Conclusion:**

Pain management in hospitalized children is ineffective, from initial assessments to reassessments after interventions, with prioritization of medication actions guided by professional judgment in the face of pain complaints.

## RESUMO

**Objetivo:**

Caracterizar o manejo da dor em crianças hospitalizadas.

**Método:**

Estudo observacional do tipo transversal, com abordagem quantitativa, de caráter retrospectivo e descritivo, realizado em um Hospital secundário do Município de São Paulo, por meio de análise de 1.251 prontuários de crianças internadas na divisão pediátrica. Os dados foram tabulados e analisados por meio de estatística descritiva.

**Resultados:**

Um total de 88,8% das crianças foi avaliado para dor com instrumentos padronizados e 86% tinham analgesia prescrita. Dentre as avaliações, 37,8% das crianças apresentaram dor; dessas, 26% apresentaram dor intensa, maior nas afecções ortopédicas; 18,3% não foram medicadas, mesmo com presença de dor e analgesia prescrita; 4,3% não tinham analgésicos prescritos; apenas 0,4% receberam medidas não farmacológicas e 40,3% tinham registro de reavaliação. Os profissionais propiciaram maior analgesia a crianças com afecções cirúrgicas e ortopédicas em comparação às afecções clínicas (p < 0,05).

**Conclusão:**

O manejo da dor em crianças hospitalizadas mostra-se ineficaz, desde as avaliações iniciais até as reavaliações após intervenções, com priorização de ações medicamentosas guiadas pelo julgamento profissional frente a queixa álgica.

## RESUMEN

**Objetivo:**

Caracterizar el manejo del dolor en niños ingresados.

**Método:**

Estudio observacional del tipo transversal, con abordaje cuantitativo, de carácter cuantitativo, de carácter retrospectivo y descriptivo, realizado en un Hospital secundario de la ciudad de São Paulo, por medio de análisis de 1.251 prontuarios de niños ingresados en el sector pediátrico. Los datos fueron tabulados y analizados por medio de estadística descriptiva.

**Resultados:**

un 88,8% de los niños fueron evaluados para dolor con instrumentos patrón y un 86% tenían analgesia prescripta. En las evaluaciones, un 37,8% de los niños presentaron dolor; de ésos un 26% presentaron dolor intenso, superior en las afecciones ortopédicas; un 18,3% no fueron medicados, incluso con presencia de dolor y analgesia prescripta; un 4,3% no tenían analgésicos prescriptos; sólo un 0,4% recibieron medidas no farmacológicas y un 40,3% tenían registro de reevaluación. Los profesionales propiciaron analgesia superior a niños con afecciones quirúrgicas y ortopédicas en comparación a las afecciones clínicas (p < 0,05).

**Conclusión:**

El manejo del dolor en niños ingresados demuestran ser ineficaces, desde las evaluaciones iniciales hasta las reevaluaciones tras intervenciones, con prioridad de acciones farmacológicas gestionadas por el juzgamiento profesional frente a la queja álgica.

## INTRODUCTION

In recent years, several agencies have dedicated themselves to the study of pain, considered one of the main complaints and disabilities worldwide, including in children^(1–4)^. To give visibility to the theme as a relevant public health issue^(1,5)^, pain control is now considered the fifth vital sign^(6–8)^. However, although the science of pain management has advanced, the difficulty of translating the knowledge generated by the evidence into clinical practice is observed in daily care, especially in sick children^(5)^.

The hospitalized child is exposed to stressful experiences, either due to disease symptoms, and/or invasive and/or painful procedures carried out as part of the treatment routine^(9,10)^. In a European investigation, of 579 children evaluated, 87% had pain complaints in one day in the hospital^(8)^, but despite pain being frequent in this population, its management is inadequate and children continually experience unrelieved pain^(11,12)^.

Pain management is understood here as stages of assessment, strategy planning, interventions – both pharmacological and non-pharmacological, and reassessments, a process that shall be cyclical for better management^(2–4)^. This management is considered as one of the most significant care functions that pediatric nurses face in the clinic^(13)^, by considering factors that can influence the characterization of pain by the child, such as age, neuropsychomotor development, behavior, and cognitive abilities^(8,14)^. To assist in this process, the use of instruments addressing multidimensional aspects, including behavioral and physiological indicators of pain^(15)^ is recommended, such as NIPS (Neonatal Infant Pain Scale), FLACC (Face, Legs, Activity, Cry and Consolability), Wong baker FACES® Pain Rating Scale (FACES), Verbal Numerical scale (gold standard)^(14)^, and COMFORT scale^(4,16)^.

Despite the existence of the aforementioned instruments and, in some services, protocols for pain management, more than half of hospitalized children experience severe unrelieved pain^(16)^. This demonstrates that scientific evidence is not being applied in clinical practices, with incompatibility between what the health sectors support and what is carried out by working professionals^(13)^, reflecting the low theoretical-practical training. In an investigation carried out in Spain, with 191 health professionals, 50% reported not having received any training in caring for children with pain and 80% recognized gaps in their knowledge regarding pain management^(17)^, which becomes a barrier to individualized care.

There are other barriers mentioned in the literature, such as lack of protocols; insufficient time for premedication in painful procedures; conflicts within the multidisciplinary team, delay in the availability of drugs; staff concerns about side effects; low confidence in instruments; lack of continuing education; parents’ reluctance to the administration of medication, among others^(6,11)^.

Inadequate pain management can lead to short-term impacts on the child’s biopsychosocial development and the experience of hospitalization by family members, as well as in the long term, with impaired sensitivity to later events that cause pain, and increased stress with traumas related to the health service^(11,17)^. Nurses play a vital role in children’s experience of pain, since they are the professionals who spend more time with the patient and are responsible for pain management, being able to change the aforementioned context with quality care or to favor traumas and stresses resulting from the unrelieved pain complaint^(6)^.

Thus, the following concern emerged: “How is pain management performed in hospitalized children, considering the whole hospitalization process?” Knowing the subject is essential, both for the training of health professionals and for the implementation of care strategies aimed at a humanized, individualized, and comprehensive therapeutic plan. Thus, this study aimed to characterize pain management in hospitalized children.

## METHOD

### Design of Study

This is an observational, cross-sectional, retrospective and descriptive study, with a quantitative approach. To guide the methodology of this study, the instrument Strengthening the reporting of observational studies in epidemiology (STROBE) was used^(18)^.

### Population

We analyzed the medical records of 1,251 hospitalized children, aged between 28 days and 14 years, 11 months and 30 days, who were in one of the sectors of the pediatrics department of the participating institution, in a period of one year.

### Local

The study was held in a secondary school teaching hospital in the city of São Paulo-SP, from July 2016 to July 2017. This service consists of 178 active beds, distributed among adult, child, and newborn health units. Clinical and surgical care for the pediatric population takes place in the pediatric department that consists of the following settings: 1) Children’s Emergency Room (*PSI*), consisting of 10 observation beds and 1 emergency room; 2) Pediatric Inpatient Unit (*UIP*), with 15 total beds, and 3) Pediatric Intensive Care Unit (PICU), with 5 available beds and 1 spare bed.

### Selection Criteria

Inclusion criteria were: medical records of children aged between 28 days and 14 years, 11 months and 30 days, hospitalized in the pediatric division, within a period of one year; and the exclusion criteria were: medical records unavailable due to use in outpatient follow-up consultations, readmissions, and use by other investigators, or which were not completely filled, with lack of information regarding pain assessment, both in filling out of the standardized instrument by the department and in the nursing notes. In the case where the child had more than one hospitalization in the established period, the records of the most recent hospitalization were considered.

### Data Collection

Data were collected through consultations and analysis of medical records, covering sociodemographic characteristics, length of stay and diagnosis, and pain management through assessment using validated instruments, pharmacological and non-pharmacological measures, and nursing diagnosis. Regarding pharmacological measures, prescription analgesics were prescribed on a regular basis, “at the discretion of the physician (ADP)” or “if necessary (Y/N)”. Regarding the nursing diagnosis, the diagnosis Acute pain (NANDA-00132) was adopted in nursing prescriptions. The study aimed to assess pain management on an ongoing basis, valuing pain trajectory during the child’s hospitalization in the pediatric department (*PSI*, *UIP*, PICU), with sequential inclusion of records with a single medical record and its clinical condition, without evaluating variables such as sedation, intubation and hospitalization sector at the time of pain.

Regarding the pain score, the institutional pain record form was consulted. This form is used in all sectors of the pediatric department, and contains the scales: 1) NIPS, indicated for newborns and children up to 2 months, with assessment of facial expression, cry, breathing patterns, arms, legs, and state of arousal; 2) FLACC, used in children aged between 2 months and 7 years and with neuropathies, assessing the face, legs, activity, cry, and consolability; 3) FACES, use in 3-year-old children, and 4) Numerical, standardized by the WHO and indicated as the gold standard for assessment, with use in children over 7 years of age, taking self-report as an assessment. The scales scores are evaluated as severe, moderate, mild pain, and no pain; FACES and Numerical include the parameter of unbearable pain. All scales have been validated for use in Brazil, and professionals from the pediatric department received training at the time of the instrument implementation.

All records made by professionals were evaluated, but the score with the greatest pain intensity was considered, that is, if the child presented mild to severe pain complaints throughout the period, the score with the greatest intensity was considered. For data from different scales to become comparable, the highest recorded score was divided by the maximum value of the corresponding scale, respecting the variation of each instrument.

In the period of one year, 1,728 hospitalizations were recorded, but 477 medical records were excluded due to the previous criteria, resulting in 1,251 medical records, read in full. Following reading, the medical diagnoses were grouped into 11 specialties: 1) Respiratory; 2) Surgical; 3) Orthopedic; 4) Metabolic; 5) Neurological; 6) Nephrological; 7) ENT; 8) Infectious; 9) Digestive; 10) Hematological, and 11) Other conditions (fever, foreign body aspiration, lupus, urticaria, and burns).

### Data Analysis and Treatment

Data were tabulated electronically and analyzed with descriptive statistics using the Software R3.5.3. Continuous variables were presented as average and categorical variables as frequency and percentage. To analyze the associations between the independent and dependent variables, the tests of Person’s, Chi-Square, Kruskal-Wallis Rank Sum, Kruskal Wallis Chi-Squared and Fisher’s exact were applied. The level of statistical significance adopted was 5% (p < 0.05).

### Ethical Aspects

The study was submitted and approved by the Ethics Committees of the Nursing School of Universidade de São Paulo, opinion No. 2.240.511, and of the Co-Participating Institution, opinion No. 2.277.191, both from 2017. The ethical principles of research based on Resolution No. 466/12 of the National Health Council were followed. Data from the medical records were collected through the formalization of the Term of Commitment.

## RESULTS

A total of 1,251 medical records were fully read. Of the total sample, 57.4% were male, the mean age was 3 years and 6 months, the main causes of hospitalization were respiratory (61.4%), surgical (16.8%) and orthopedic (3.8%) conditions. The length of stay ranged from 3 to 10 days, with a mean of 5 days, with 88.8% being assessed for pain using the following scales: NIPPS (5.6%), FLACC (73.3%), FACES (3.5%) and Numerical (21.3%), and 481 had pain at some point during hospitalization. Although the team received previous training on the scales, 11.2% of the children did not have their pain evaluated by them, without justification in the medical records. Table 1 characterizes children and pain assessment in the total sample.

**Table 1.** Sample characteristics (N = 1251) – São Paulo, SP, Brazil, 2019.

	Mean (min – max)	95% CI
**Age (years)**	**3.6 (28 days–14 years)**	**–**
**Sex**	**N (%)**	**95% CI**
Female	533 (42.61)	[39.89–45.36]
Male	718 (57.39)	[54.64–60.11]
**Causes of hospitalization**	**N (%)**	**95% CI**
Respiratory	762 (61.40)	[58.66–64.07]
Surgical	209 (16.84)	[14.86–19.03]
Orthopedic	48 (3.87)	[2.93–5.09]
Infectious	39 (3.14)	[2.31–4.27]
Neurological	38 (2.26)	[1.57–3.24]
Nephrological	33 (2.66)	[1.90–3.71]
Digestive	29 (2.34)	[1.63–3.34]
ENT	21 (1.69)	[1.11–2.57]
Hematological	20 (1.61)	[1.05–2.48]
Metabolic	14 (1.13)	[0.67–1.88]
Others	38 (3.06)	[2.24–4.17]
**Length of stay (days)**	**Mean (min – max)**	**95% CI**
Respiratory	4.68 (0–305)	[3.0–3.5]
Surgical	5.52 (0–366)	[2.5–3.5]
Orthopedic	3.0 (1–23)	[1.5–3.0]
Infectious	7.36 (1–94)	[3.0–5.5]
Neurological	5.46 (1–37)	[3.0–5.5]
Nephrological	3.97 (1–11)	[3.0–4.0]
Digestive	4.41 (1–30)	[2.0–3.5]
ENT	4.57 (0–12)	[3.0–6.5]
Hematological	3.4 (1–9)	[2.5–4.0]
Metabolic	8.21(3–14)	[6.0–10.5]
Others	10.05 (0–280)	[2.0–4.0]
**Presence of pain**	**N (%)**	**95% CI**
Yes	481 (38.7)	[36.03–41.44]
No	761 (61.3)	[58.56–63.97]
**Use of validated instrument for pain assessment**	**N (%)**	**95% CI**
Yes	1,172 (88.8)	[32.41–38–28]
No	79 (11.2)	[50.22–51.45]
Scale Used*	**N (%)**	**95% CI**
NIPPS (Newborns to months)	70 (5.63)	[4.48–7.06]
FLACC (2 months to 7 years and neuropathic patients)	912 (73.37)	[70.83–75.75]
Faces (3 years)	44 (3.52)	[2.65–4.72]
Numerical (>7 years)	264 (21.32)	[19.13–23.68]
**Nursing Diagnosis: Acute pain**	**N (%)**	**95% CI**
Yes	375 (30.1)	[27.61–32.7]
No	871 (69.9)	[67.3–72.39]

*The same scale was used more than once by the same patient.

In the total sample, 86% of the children had medication prescribed for pain management (Table 2). Regarding children with pain, 95.6% had analgesics prescribed, totaling 774 medications, and 81.7% were medicated, with the most used medications being dipyrone (76.1%), paracetamol (22.8%), ketoprofen (16.4%), and tramal (13.1%).

**Table 2.** Description of drugs prescribed – São Paulo, SP, Brazil, 2019.

Medicines*	Total number of children (N = 1251)	Children with pain (N = 481)	
	Prescribed N (%)	Prescribed N (%)	Administered N (%)
Dipyrone	1015 (81.1)	428 (88.9)	366 (76.1)
Paracetamol	213 (17.1)	118 (24.5)	110 (22.8)
Ketoprofen	102 (8.1)	82 (17)	79 (16.4)
Tramal	77 (6.1)	64 (13.3)	63 (13.1)
Ketorolac	48 (3.8)	35 (7.3)	34 (7.1)
Ibuprofen	40 (3.2)	21 (4.3)	19 (3.9)
Morphine	18 (1.4)	18 (3.7)	18 (3.7)
Codeine	2 (0.1)	1 (0.2)	1 (0.2)
Gabapentin	2 (0.1)	2 (0.4)	2 (0.4)
Xylocaine Spray	2 (0.1)	1 (0.2)	1 (0.2)
Simethicone	1 (0.07)	0 (0)	0 (0)
Buscopan	1 (0.07)	1 (0.2)	1 (0.2)
Xylocaine Gel	1 (0.07)	1 (0.2)	1 (0.2)
Dolantine	1 (0.07)	1 (0.2)	1 (0.2)

*Some children had more than one medication prescribed and administered.

Children with respiratory (46.1%), surgical (24.9%), and orthopedic (6.2%) conditions were the ones with the most pain. In terms of intensity, 26% of the children had severe pain, more prevalent in orthopedic (63.4%) and surgical (55.8%) conditions. Children with orthopedic and surgical conditions had the highest average number of drugs prescribed (Table 3).

**Table 3.** Pain management by specialty – São Paulo, SP, Brazil, 2019.

Presence of pain = 481 children						
Specialty	Presence of pain	Pain intensity			Medicines	
					Prescribed	Administered
	N (%)	Mild N (%)	Moderate N (%)	Severe N (%)	Mean (min – max)	N (%)
Respiratory	222 (46.1)	148 (66.6)	58 (26.2)	16 (7.2)	1 (1–4)	159 (71.6)
Surgical	120 (24.9)	20 (16.6)	33 (27.6)	67 (55.8)	2 (1–5)	112 (93.3)
Orthopedic	30 (6.2)	5 (16.6)	6 (20.0)	19 (63.4)	3 (1–6)	28 (93.3)
Infectious	16 (3.4)	5 (31.2)	7 (43.8)	4 (25,0)	2 (1–5)	13 (81.2)
Neurological	10 (2.0)	3 (30.0)	2 (20,0)	5 (50,0)	2 (1–4)	10 (100)
Nephrological	20 (4.0)	9 (45.0)	10 (50.0)	1 (5,0)	2 (1–5)	17 (85)
Digestive	18 (3.6)	8 (44.4)	7 (38.9)	3 (16.7)	2 (1–4)	14 (77.7)
ENT	12 (2.4)	9 (75.0)	2 (16.6)	1 (8.4)	2 (1–3)	10 (83.3)
Hematological	11 (2.8)	3 (27.2)	4 (36.4)	4 (36.4)	1 (1–3)	9 (81.8)
Metabolic	7 (1.4)	2 (28.6)	2 (28.6)	3 (42.8)	1 (1–3)	7 (100)
Others	15 (3.2)	9 (60.0)	4 (26.7)	2 (13.3)	2 (1–3)	14 (93.3)

Some children did not undergo interventions for analgesia, despite having pain identified by the scales and having medication prescribed. The absence of analgesia, even with the medication prescribed, was observed in 18.3% of the children, being more frequent among hospitalizations for clinical causes (28.4% in respiratory, 22.3% in digestive, 18.8% in infectious, 18.2% in hematological, 16.7% in otorhinolaryngological, 15% in nephrological diseases, and 6.7% in other conditions), compared to hospitalizations for surgical (6.7%) and orthopedic (6.7%) causes. Of the 18.3% children with no administration of analgesia, 84% had mild pain, 9% moderate pain, and 7% severe pain. And 4.3% of children with pain had no drug prescribed.

Through the application of statistical tests to compare the variables, it was possible to observe that children of the surgical specialty were the most medicated, compared to metabolic (p < 0.001), hematological (p < 0.003), infectious (p = 0.01), nephrological (p = 0.01), orthopedic (p < 0.05), respiratory (p < 0.001) disorders, and other conditions (p < 0.001). And orthopedic conditions were more medicated compared to otorhinolaryngological respiratory and other conditions, with p < 0.001.

In addition to pharmacological pain management, there is also non-pharmacological management, but only 6 children (0.4%) received non-pharmacological measures recorded in the nursing notes, namely: local heat (50%), distraction (33.3 %), and cold compress (16.7%). It should be noted that among the 18.3% with no analgesia performed, none of them received non-pharmacological measures for pain management, according to the notes.

The nursing diagnosis NANDA 00132-Acute pain was applied to 30.1% of hospitalized children (Table 1), and was linked to the nursing activity prescribed by the nurse: “To perform a comprehensive pain assessment that includes the location, characteristics, onset, duration, frequency, quality, intensity, or severity of pain, and precipitating factors.” Establishing the relationship between the presence of pain and the choice of this diagnosis, it was observed that it was adopted for 53.8% of the children who had pain at some point during care, with a relative risk of 3.63. And it was chosen for 14.8% of the children who did not have pain at any time during care.

The assessment instrument standardized by the pediatric department does not offer a specific field for recording pain reassessments, which are made in the nursing notes. However, of the children with pain, only 40.3% had a record of reassessments in these notes.

## DISCUSSION

In this study, data covered the entire pain trajectory experienced by children, allowing for a comprehensive view related to various aspects within the hospitalization experience. In the data collection setting, there was no institutional protocol to guide practices regarding pain complaints, only an instrument that standardizes pain assessment with validated scales, in which, routinely, since admission to the *PSI*, they are evaluated in a systematized and continuous way between its transition in the sectors, every 4 hours, as recommended by the World Health Organization (WHO)^(2–4)^.

The first step in pain management is assessment, which is important for successful treatment, as children whose pain complaints are evaluated and documented are more likely to receive treatment. Nurses report that this stage is challenging, as the use of an appropriate instrument is necessary, which requires theoretical knowledge about concepts of pain and child development^(19)^.

Investigations report that institutions with structured programs for pain management generally achieve better quality in this process^(13)^; however, failures are observed in the literature, as seen in this study, in which, even with standardization and systematization of care, 11.2% of the total sample were not assessed for pain. This low assessment corroborates a study carried out at the Johns Hopkins Hospital with 199 children, which, although 86% reported pain, only 48% had a pain score documented in their medical records^(5)^.

In addition, the assessment needs to be carried out properly, but it is noted that the scales are used mechanically, which leads to non-representative pain scores. In a Brazilian investigation carried out in a Neonatal Intensive Care Unit, it is observed that in the period of 3 days, 90 newborns underwent 2732 painful procedures, but there were only 1257 reports in medical records with validated scales, and of these only 5.6% corresponded to the presence of pain^(15)^. In a study carried out in Israel with 82 pediatric nurses, 75% reported that they rarely or never used validated scales, but documented pain complaints in other places. When asked about the reason for low adherence, 31% reported difficulties in use^(19)^.

In a qualitative study carried out in Sweden, nurses reported that they did not use scales to assess pain due to low confidence, the belief that they did not provide a comprehensive assessment, and the view that their use was an extra workload. It is worth mentioning that these professionals recognized that they neglected the integral assessment, reporting that they would adopt it if there was a standardization in the routine^(8)^; however, in this study, this pattern is observed, but not assessment adherence and performance of quality.

Even with the use of validated scales, pain assessment is subjective, health professionals observe behavioral and physiological aspects and their judgment on a given variable indicates the score^(10,16)^, and in the case of the numerical verbal scale, considered the gold standard, the pain complaint may not be valued^(13)^. An American study illustrates the above, in which 48.2% of 178 users assessed reported severe pain, but only 15% of professionals documented this score^(7)^. As the score depends on professional judgment, there is a risk of him/her adopting a more accommodated and mechanical posture, and failing to explore important aspects for taking action. Having the assessment implemented as the fifth vital sign, for example, does not guarantee that the assessments fully explore the meaning of the painful experience, if the professional is not well prepared to perform the pain assessment^(13)^, according to the hypotheses of some nurses^(8)^.

In this study, more than one validated scale was chosen for the same child. This can take place in cases where the condition worsens, reducing the ability to understand and respond to pain assessment. Conversely, there is the possibility that the nurse chooses the pain assessment scale inappropriately, due to the lack of specific knowledge on the subject or difficulty in recognizing specific characteristics of the child, which leads to a subjective assessment, with difficult comparison over the course of time, not allowing to assess a progression of the child’s condition^(8)^.

Considering that the assessment is carried out carefully and the presence of pain is detected, it is expected that in an agile and cautious way, a care plan will be structured that meets the child’s needs, with the use of interventions, both pharmacological and non-pharmacological assertively. The American Academy of Pediatrics and the WHO recommend the association between these measures so that pain management occurs more appropriately and emphasize that non-pharmacological measures may be more effective than isolated medications^(2–4,20)^.

In this study, it is observed that in children with pain confirmed by scales, 95.6% had drugs prescribed, but only 81.7% were medicated, which shows that there is still a hesitation on the part of professionals in the child’s medicalization. When comparing this age group with adults, even though both have similar diagnoses, children receive less analgesia, and the younger they are, the less likely they are to have adequate analgesia in the medical environment^(12)^. This aspect is observed in a Brazilian investigation, in which of 2736 painful procedures in neonates, only 216 of these were performed with the aid of drugs^(15)^. This problem also occurs at the time of prescription of analgesics, either due to low training, low priority given to pain (p < 0.001)^(6)^, as well as the insecurity in prescribing as pediatric drugs are *off label*, that is, not tested in the pediatric population with low evidence regarding their use^(1,11)^.

Professionals often opt for medications from the non-steroidal anti-inflammatory class to treat pain^(7)^, as seen in this study, even when there is a report of severe pain. The World Health Organization recommends, through the analgesic ladder^(3)^, that treatment of severe pain is done with the association of analgesics, contemplating opioids and adjuvants; however, in this study, it is observed that even with 25.9% reporting severe pain, only 3.7% used morphine, 0.4% codeine, and 0.4% Gabapentin. In an investigation in China with 211 nurses and 45 physicians, with both professionals working in Pediatrics, 55.7% of nurses and 66.7% of physicians were hesitant to give opioids to children^(14)^, corroborating the report of another study in which, of 199 children, less than 25% were prescribed opioids, despite the pain being classified as severe^(5)^.

In the international literature, children with surgical^(21,22)^ and orthopedic^(12,23)^ conditions have better management of pain complaints, which can influence the assessment of health professionals who, by taking into account their previous experiences, tend to prioritize these specialties, while under-evaluating and under-treating conditions that they believe do not cause pain. This study corroborates what has been exposed here, that surgical and orthopedic conditions were more medicated for pain compared to other clinical specialties (p < 0.05), despite these latter having documented pain complaints and medications prescribed. So it is worth reflecting: Why are children with pain validated by specific instruments still undertreated?

Another point, which exemplifies the argument, is that 4.3% of children with pain did not have any drug prescribed and did not undergo any non-pharmacological intervention. Similar aspects were observed in a study with neonates subjected to 2736 painful procedures, in which 80.2% did not receive non- pharmacological measures that could be applied^(15)^. In this study, few non-pharmacological measures for pain relief were observed, according to medical records, although it is perceived in clinical practice, but with poor records. The institutional form did not offer a field for recording such behaviors and this may favor failures, as it implies the need to record it in another form, such as in this study that 1.2% of the children who underwent these measures had the record found in the nursing notes. This finding corroborates audit studies, which demonstrate irregular documentation^(13)^.

Considering the period of permanence of the nursing team with the patient, the application of non-pharmacological measures can be a frequent work tool, but resistance is still present. In a Chinese study carried out with 211 nurses and 45 physicians, 12.6% of nurses and 11.1% of physicians believed that non- pharmacological management for pain relief was inadequate and, consequently, did not use it. Although this study reported that participants received training in pain management previously^(14)^, this finding suggests that, despite the evidence on the subject representing a considerable percentage of scientific publications, some difficulty is observed in transferring this knowledge to clinical practice^(24)^.

Professional attitudes towards a certain action still guide practice. In a study carried out in Ethiopia^(9)^, with 169 nurses, 35.5% had a favorable attitude towards the use of non- pharmacological measures for pain relief, being 3 times more likely to use them. Furthermore, although the Chinese study^(14)^ demonstrated that even with training there was still resistance, in this Ethiopian investigation^(9)^ 14.2% had training in the use of measures, and this proved to be a positive predictor for their use.

It is relevant that non-pharmacological interventions are integrated within nursing practice, for which one possibility is the use of the nursing process. In this study, 30.1% of the total sample and 55.8% of children with pain had the nursing diagnosis NANDA 00132- Acute Pain. This use can guide the prescription of activities for the technical team and enhance the use of non-pharmacological measures. It is worth mentioning that a considerable number of children with pain reports did not have the diagnosis raised, requiring greater nurse encouragement for their use.

Pain management is carried out with cyclical steps, and reassessment is the most important aspect as it will assess the effectiveness of management strategies, the care plan and, consequently, the need for changes to provide adequate relief^(14)^. However, in this study, only 40.3% of children with pain had their pain reassessed, given the absence of these records; it is implied that there is a break in the flow of actions that would favor the relief of pain in hospitalized children. This failure may demonstrate the lack of technical knowledge from the team and the lack of recognition of the sustainability of the pain management process.

For all pain management, the expertise of those who carry out the steps with the intention of proposing an assertive action plan is essential. Nevertheless, according to previously demonstrated findings, there is little investment in professionals’ training on pain, which directly reflects on clinical practice. In an investigation in Africa that evaluated the knowledge of 180 nurses in pain management, 38.9% showed unsatisfactory knowledge regarding pain assessment and the use of analgesics in children, despite receiving an overall good score^(11)^. In addition to the above, there are several other barriers^(6)^, but identifying them is not the only way to change the current reality, the creation of strategies is required.

One possibility is the structuring of institutional protocols for pain management, built together with the team that provides care to the child, which favors the understanding of the real needs of both. In Spain, of 191 health professionals, 96% recognized the need to formulate protocols^(17)^. In Africa, 11 pediatric nurses were evaluated after implementing a protocol, and 97.1% positively changed their practices^(11)^. In Canada, after the implementation of an institutional protocol for pain management in 16 hospital units, with follow-up at 3 years, it was observed that, in fact, pain was more assessed with validated instruments (p = 0.01) and there was an increase in the use of analgesics (p = 0.04); however the process did not remain constant and the professionals stopped strictly following the recommended steps and performed the process mechanically, with management guided by their judgment with beliefs and values^(25)^.

Thus, it is observed that institutional protocols are positive predictors for change in the context of pain assessment^(11,25)^. However, besides it, improvement of the team responsible for pain management, with continuing education, based on strategies focused on making pain important, understood, visible, and better managed is required^(1,14)^. Professionals’ expertise can be the key to best practices, but performing this role requires technical-scientific knowledge and daily effort to maintain consistency in this process^(25)^. In an American study assessing the implementation of team improvement strategies in terms of pain management, continuing education was shown to be a positive predictor of improving team practices^(6)^.

There is an influence of personal beliefs and values in the conduction of pain management. The implicit power relationships between health professionals and children and among the various members of all health teams permeate the dynamics of care within the institution, and can influence in more assertive decision-making regarding pain control. Thus, it becomes relevant that the cited practices are included and recognized due to the importance of management within the institutional culture and that possibilities for team integration are provided. With the readaptation of practices, hospitalized children can have the experience in a context where pain exists, but its relief can be managed.

The main limitations of this study were the lack of data collection referring to the inpatient unit and the child’s clinical condition; the inadequacy of the institutional pain assessment instrument filling; and the reduced record of actions performed for pain management by nursing professionals.

## CONCLUSION

This study demonstrates that pain management in hospitalized children is still ineffective. The assessment, even if standardized by the co-participating institution, is not fully carried out. Interventions have a pharmacological focus, health professionals provide better management of surgical and orthopedic conditions compared to clinical specialties, despite the fact that these latter have pain complaints. Non-pharmacological measures are rarely performed, as is reassessment after interventions.

Based on knowledge about the profile of children with pain, the possibility arises of adopting, together with the multidisciplinary team, an approach that allows comprehensive but individualized pain relief actions, according to each clinical specialty, recognizing needs, with integration of the subjectivity and stage of the child’s development in a careful evaluation that allows the elaboration of an individual and humanized care plan. The need to formulate an institutional protocol for pain management is highlighted, with continuing education for the professional team’s improvement, to make pain important, understood, visible, and better managed.

## ASSOCIATE EDITOR

Ivone Evangelista Cabral
